# From Learner to Provider: Navigating Role Tensions in Postgraduate Medical Training Through Activity Theory

**DOI:** 10.5334/pme.1499

**Published:** 2025-02-14

**Authors:** Sin-Yee Patty Kwong, Shiuan-Ruey Yu, Kuo-Chen Liao, Shu-Chen Liao, Cheng-Ting Hsiao, Chung-Hsien Chaou

**Affiliations:** 1 Chang-Gung Medical Education Research Centre, Chang Gung Memorial Hospital, Taiwan; 2 Chang Gung University College of Medicine, Taoyuan, Taiwan; 3 Chang-Gung University School of Medicine, Taoyuan, Taiwan; 4 Department of Medical Education, Chang-Gung Memorial Hospital, Keelung, Taiwan; 5 Chiayi Chang-Gung Memorial Hospital, Chiayi, Taiwan; 6 Chang Gung University School of Medicine and physician, Taiwan; 7 Department of Emergency Medicine, Chang Gung Memorial Hospital, Linkou, Taiwan

## Abstract

**Introduction:**

The transition from medical school to residency, especially during the postgraduate year (PGY) internship, poses unique challenges as graduates navigate clinical practice complexities. Understanding PGYs’ experiences is crucial for developing effective support strategies to promote their professional growth and well-being.

**Methods:**

This qualitative, longitudinal study followed ten PGYs from August 2021 to July 2023, using biannual audio diary based on open-ended questions to capture their experiences. Data analysis, guided by Activity Theory, focused on role conflicts and contradictions as PGYs transitioned from learners to practicing physicians.

**Results:**

The analysis revealed prevalent role conflicts and contradictions, primarily due to the tension between the PGYs’ roles as learner and healthcare provider. Differences in objectives between PGYs and practicing doctors further exacerbated these conflicts, leading to clashes in priorities and care approaches. Consequently, PGYs experienced reality shock, lack of confidence, and feelings of incompetence, compounded by heavy workloads and exhaustion. These findings underscored the need for support and resources to help PGYs navigate these challenges and succeed in their healthcare roles.

**Discussions:**

Using Activity Theory to analyze the inherent challenges and contradictions within the PGY experience, this study offers insights for enhancing PGY preparedness, fostering both professional development and well-being. Drawing on recommendations supported by existing literature, which are stratified by tools, rules, and division of labor, we propose targeted strategies to address specific facets of the PGY role, thereby improving the overall training environment. This research highlights the need for tailored interventions to support PGYs through the challenging transition into clinical practice.

## Introduction

Medical education is a profound journey characterized by rigorous training, hands-on learning, and the gradual evolution from novice to proficient practitioner. At the outset of their medical journey, aspiring doctors encounter a diverse array of academic, clinical, and personal challenges that shape their journey towards becoming competent healthcare professionals. Throughout medical school and residency training, they navigate the complexities of medical knowledge, clinical skill acquisition, and professional growth [1,2,3].

Central to this journey lies the postgraduate rotation stage, often referred to as postgraduate year (PGY) internship, a pivotal phase where medical students delve into clinical practice under the guidance of experienced professionals [4]. This transitional period not only signifies the commencement of hands-on patient care responsibilities but also serves as a crucible for shaping future medical practitioners. Although supervision is available during PGY, new doctors in these post-graduate periods are frequently expected to independently practice clinical skills and make decisions, a situation that international research highlights as highly stressful [5,6,7]. Factors contributing to this stress include inadequate preparation during medical school and insufficient support and education for newly qualified doctors as they enter clinical practice [7]. This stress can lead to burnout, which is a significant factor contributing to medical errors and poses serious risks to patient safety [8]. Studies on resident burnout also show its negative impact on work performance and the effectiveness of resident education, potentially threatening the quality of patient care [9,10,11,12].

The PGY internship is a focal point for examination for several reasons. It represents a critical juncture in medical training, bridging the gap between theoretical knowledge acquired during medical school and the practical skills essential for autonomous clinical practice [13]. PGY trainees actively engage in clinical settings, where they navigate complex patient encounters, diagnostic dilemmas, and treatment decisions under supervision [14]. The internship period is filled with diverse possibilities and uncertainties, making it a unique opportunity to explore the intersection of education and practice. The PGY internship serves as a microcosm of the broader medical education system, reflecting the impact of curriculum reforms, pedagogical approaches, and institutional practices on trainees’ learning experiences and professional development. Despite the pivotal role of PGYs in medical education, limited research exists on the organizational or team aspects of accommodating PGYs on rotations, and the need for research on an overarching conceptual model to create more theory-driven results was suggested [15]. This study seeks to explore the PGY journey utilizing Activity Theory as an analytical framework [16]. We aimed to explore the complex dynamics within the activity systems, examining interactions and contradictions among trainees, supervisors, patients, and institutional structures.

## Theoretical Framework

### Activity Theory

Activity Theory (AT), also known as Cultural-Historical Activity Theory (CHAT), originated from the works of Vygotsky and Leont’ev and was further developed by Engeström, presenting a robust framework for analyzing human activities within sociocultural contexts [17]. Initially formulated to understand the development of human consciousness, Activity Theory has found extensive application in various fields, including medical education. Engeström posits that Activity Theory perceives human activities as purposeful actions carried out by individuals within a social environment [17,18]. Within the Activity Theory framework, an activity system forms the basic unit of analysis and is oriented towards an object, which embodies the long-term purpose of the activity and generates horizons for possible actions [18,19]. Engeström further developed AT by integrating interactions among adjacent activity systems, revealing contradictions and tensions that provide insights into cultural and social factors influencing learning outcomes [16].

In educational contexts, Activity Theory provides a lens through which to analyze learning processes, considering the complex interactions among learners, teachers, tools, and sociocultural environments. Numerous scholars have applied Activity Theory to analyze learning experiences across diverse educational settings [20]. Activity Theory proves beneficial not only for understanding current practices but also for anticipating how practice and learning might evolve within dynamic systems, making it particularly suitable for investigating practice-based learning [18,19,21,22]. In the current study, Activity Theory was chosen for its strength in capturing the complex, interactive experiences of PGY learners within the healthcare environment. Rather than focusing solely on individual perspectives, this framework allows us to examine the dynamic relationships and mutual influences among PGYs and their broader clinical context.

## Methodology

### Study setting

This qualitative study was conducted between August 2021 and July 2023, spanning over two years to thoroughly explore the PGY internship experience. This extended data collection was necessary to observe longitudinal patterns in role adaptation and identify evolving tensions between learning and practicing. The study was conducted in four major teaching hospitals in Taiwan, with overall clinical capacity of 8,600 beds and training capacity of 280 PGY doctors annually. These hospitals represent diverse healthcare environments and patient populations, contributing to the variability of the data collected. Ethical approval has been granted by Chang Gung Medical Foundation- Institutional Review Board (IRB NO. 202002344B0A3).

### Participant sampling

Taiwanese medical graduates progress through two years of a Postgraduate Years (PGY) stage following their completion of medical school. Equivalent stages of medical student training in other countries vary in nomenclature and structure. In the United Kingdom (UK), graduates enter the Foundation Programme, comprising Foundation Year 1 (FY1) and Foundation Year 2 (FY2), with FY1 akin to PGY1 in clinical responsibilities. Whereas, in the United States of America (USA), medical graduates enter residency programs post-medical school, with PGY-1 mirroring Taiwan’s PGY. In Australia, internship training follows medical school, with the intern year being parallel to Taiwan’s PGY in its timing and clinical exposure [23,24]. These PGY-equivalent stages globally serve as pivotal transitional periods, providing early-career physicians with essential clinical experience and skills before further specialization.

A cohort of ten medical graduates was recruited at the beginning of their PGY1 and followed for two years until the completion of their second year (PGY2). The PGY training for this cohort began in August 2021 and continued until July 2023. Participants were recruited through posters displayed on the hospital bulletin boards, and ten PGYs volunteered to participate, comprising four males and six females with an average age of 25. Prior to their participation, all participants provided written informed consent after being briefed on an overview of the study’s objectives and methodology.

### Data collection

Regularly recorded audio diaries serve as a potent tool for researchers to capture real-time experiences, providing valuable insights into the progression and reflections of learning over time [25]. These diaries are particularly valuable for longitudinal qualitative analysis, offering a temporal context that aids in understanding the evolution of attitudes, challenges, and insights throughout the PGY clinical experience [26]. Every participant was required to record an audio diary four times, spaced six months apart, wherein they responded to pre-defined open-ended questions regarding their learning journey. The two-year data collection aligned with the two-year PGY program to provide a longitudinal view of trainees’ development. Recording every six months allowed us to capture evolving experiences while balancing the need for detailed insights with the logistical considerations of data collection. This frequency was determined by the research team to optimize the data’s richness without overburdening participants.

A full interview guide (Appendix A) is included to clarify how the questions align with our research goals, particularly regarding role tensions. Questions were designed to elicit detailed responses about the participants’ experiences as learners and doctors. For example, participants were asked to reflect on their biggest improvement in the past two months, with the freedom to provide examples to illustrate their perceptions and sentiments. A total of 35 audio diaries were recorded by 10 participants. The completion rates for the four rounds were 100%, 90%, 80%, and 80%, respectively, reflecting participants’ consistent engagement over the course of the study. Previous literature supports this sample size as sufficient for thematic saturation, enabling a thorough exploration of role tensions [27]. Initially, all participants contributed 10 records (100%). In subsequent sessions, there were nine, eight, and eight records, respectively. These recordings were transcribed into free-flowing text and analyzed independently by two researchers (SYK and SRY).

### Data analysis

All audio recordings were transcribed verbatim, and any identifying information was removed. The transcripts were organized and categorized using the ATLAS.ti software. We utilized the process of inductive coding [28], and two members of our research team (SYK and SRY) independently reviewed the 35 transcripts. A third researcher, CHC, reviewed the codes. Next, we engaged in discussions regarding the disparities in the coding and endeavored to achieve consensus on the labeling and definitions of codes.

Following the initial inductive coding, analysis with Activity Theory (AT) was performed. This study draws on the third generation of AT, which emphasizes multiple interacting activity systems and helps identify contradictions within and between these systems. The third generation of AT extends beyond analyzing individual activity systems to examining interactions between multiple systems. This approach emphasizes how different communities or groups, each with unique rules, tools, and divisions of labor, interact and influence each other, especially when their goals and practices intersect. Key components of the activity system include the ‘subject’ aiming to bring about change, ‘tools or artifacts’ representing culturally produced means for achieving goals, ‘rules’ pertaining to norms and regulations, ‘community’ comprising individuals and subgroups sharing a common object, and the ‘division of labor’, referring to the horizontal division of tasks and the vertical division of power and status [17,29,30]. AT allowed us to construct two activity systems of the PGY internship from different perspectives, providing a conceptual framework for identifying contradictions, challenges and adaptation during the PGY learning experience.

### Reflexivity Statement

Reflexivity was practiced consistently throughout this research project. Our research team includes members from diverse backgrounds, including two research assistants with nursing experience and several senior faculty members or leaders from various medical specialties. These varied perspectives enriched our discussions and contributed to a nuanced interpretation of the data. Additionally, none of us had any prior interactions with the participants, supporting an unbiased analysis of their experiences [31].

## Results

### Mapping Activity Theory and PGY objects

**Figure 1:**
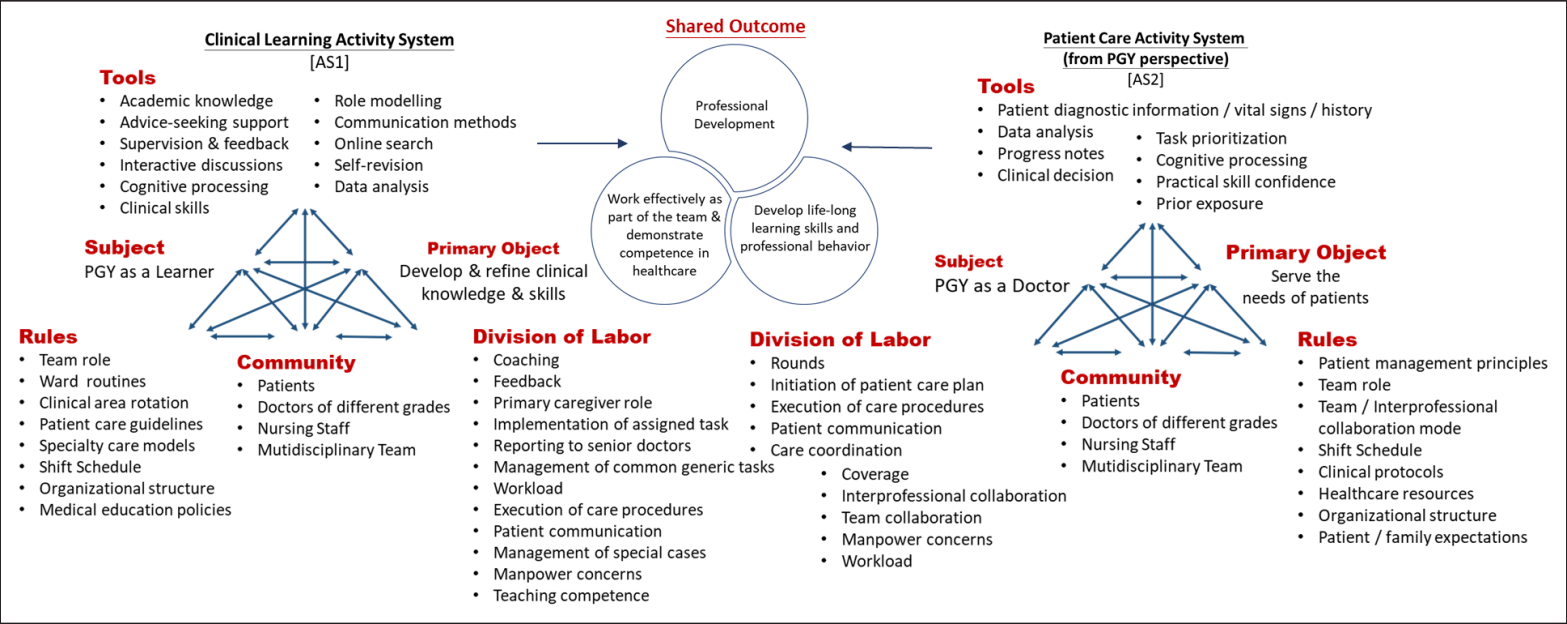
Mapping Activity Theory and PGY objects.

Initially, we aimed to use the Activity Theory as a theoretical lens to understand what activity systems are at play in shaping the learning experiences of PGY trainees and to identify the tensions and contradictions in PGY training. However, during the analysis, we noted that participants frequently expressed challenges and conflicts associated with shifting between the roles of “learner” and “doctor.” This recurrent theme highlighted their struggle to balance these two identities. As a result, we focused our analysis on these two primary roles, framing them as distinct activity systems in order to better capture the tensions and transitions PGYs experience in their development. Examining two activity systems side by side (Figure 1) can elucidate both the common aspects and contradictions between the activities, with PGYs assuming various roles, both as learners and as doctors.

In the Clinical Learning Activity System (AS1), the PGY functions primarily as a learner, with the primary object of developing and refining their clinical knowledge and skills. Conversely, in the Primary Care Activity System (AS2), the PGY’s main objective is to address the needs of patients. Despite this distinction, both systems share common outcomes, such as professional development, working effectively as part of the team, demonstrating competence in healthcare practices, and developing lifelong learning skills and professional behavior. Sample quotes supporting the components within the activity systems aid in comprehending how these activities are related to each other and mediated (The quotations are labeled as: participant number; record number; quotation number).

### Tools

Learners frequently rely on institutional resources, guidelines, and senior staff feedback. Doctors, in contrast, increasingly depend on personal experience and patient data to inform decisions. Some of the important examples are presented here:

Clinical learning activity system [AS1]: Role Modelling

‘The teacher is quite serious about teaching. During rounds, he would carefully open up lab results or images and discuss them with us in detail. He is also a very strict mentor. For example, once in the operating room, he asked how to perform an examination for a patient with hernia. Although the teacher had mentioned it before, I didn’t fully understand at the time. He scolded me, but I came to understand his strict requirements for us. From working with him this month, I feel like I’ve learned a lot about the qualities that make an excellent surgeon.’ (R3-4-2).

Patient care activity system [AS2]: Clinical decision-making

‘When children come in for chemotherapy, if they experience any physical discomfort, I prescribe them with symptom-relief medications. For example, some children feel nauseous after chemotherapy, I might promptly check if it’s a side effect of the treatment or if there are other reasons causing their vomiting. I then address the cause of their vomiting or provide antiemetic medication for symptom relief.’ (R5-4-4).

### Rules

In the learner system, rules are framed around structured learning objectives and institutional protocols. In the doctor system, these rules evolve into standards of care and ethical guidelines, with flexibility often reduced by the need for immediate, effective patient management. Some of the important examples are presented here:

Clinical learning activity system [AS1]: Team role

‘In this team, my role is more like a basic learner. My learning might involve handling relatively simple patient complaints, such as if they feel headaches or stomachaches. I can assist by making adjustments to their medications and providing some basic differential diagnoses.’ (R5-1-4).

Patient care activity system [AS2]: Team role

‘The main role is to play a resident physician. My contributions mainly include assisting with invasive procedures if needed for patients. If a patient requires urgent interventions, I can conduct preliminary diagnosis and treatment, before contact and report back to the attending physician.’ (R8-4-4).

### Division of Labor

Within the learner role, tasks are divided hierarchically, with close supervision. As interns transition to the doctor role, labor is distributed among teams with a higher expectation for independent task completion and decision-making.

Clinical learning activity system [AS1]: Coaching & learning

‘Attending physician would assign a topic, allowing us time to prepare by consulting textbooks or searching online. Then, next week, we would share with everyone what we learned or found. For any deficiencies, he would open his own lecture notes and provide further explanations, which was a learning method that I found extremely beneficial.’ (R1-1-13).

Patient care activity system [AS2]: Clinical workload

‘Within just a short span of a dozen days, I handled patients with respiratory acidosis, respiratory failure, intestinal perforation, acute cholecystitis, and myocardial infarction. For these patients, immediate blood draws, interventions, specialist consultations, urgent examinations, and real-time discussions with attending physicians were almost always required. It was also frequently necessary to explain the conditions to patient relatives in detail.’ (R1-3-12).

### Longitudinal Progression

Our longitudinal analysis focused on tracking changes in PGY participants’ experiences and skill development across the two-year span. Early recordings often highlighted challenges with time management and confidence in clinical decision-making, while later recordings showed increased autonomy and comfort in managing patient care. By comparing these points, we observed progress in participants’ abilities and professional identity formation. This progression underscores how trainees’ skills and confidence evolved over time, illustrating the impact of continuous practice and mentorship in the PGY journey.

### Role conflicts create contradictions and tensions

The purpose of using AT is to understand where conflicts and tensions lie as a basis for improved forms of activity [19,32]. One prominent conflict identified is the duality between the roles of learner and healthcare provider. This conflict, stemming from the inherent dual responsibilities, create tensions. PGYs face the challenge of balancing the imperative to provide high-quality patient care with the need to acquire the essential knowledge, skills, and experience required for their professional growth. Further exacerbating these conflicts are the differing objectives between PGY and practicing doctors, leading to potential clashes, including learning versus efficiency, learning demands versus patient care, perception of role, and supervision versus autonomy. We outlined the contradictions encountered by PGYs, accompanied by illustrative quotes in Table 1.

**Table 1:**
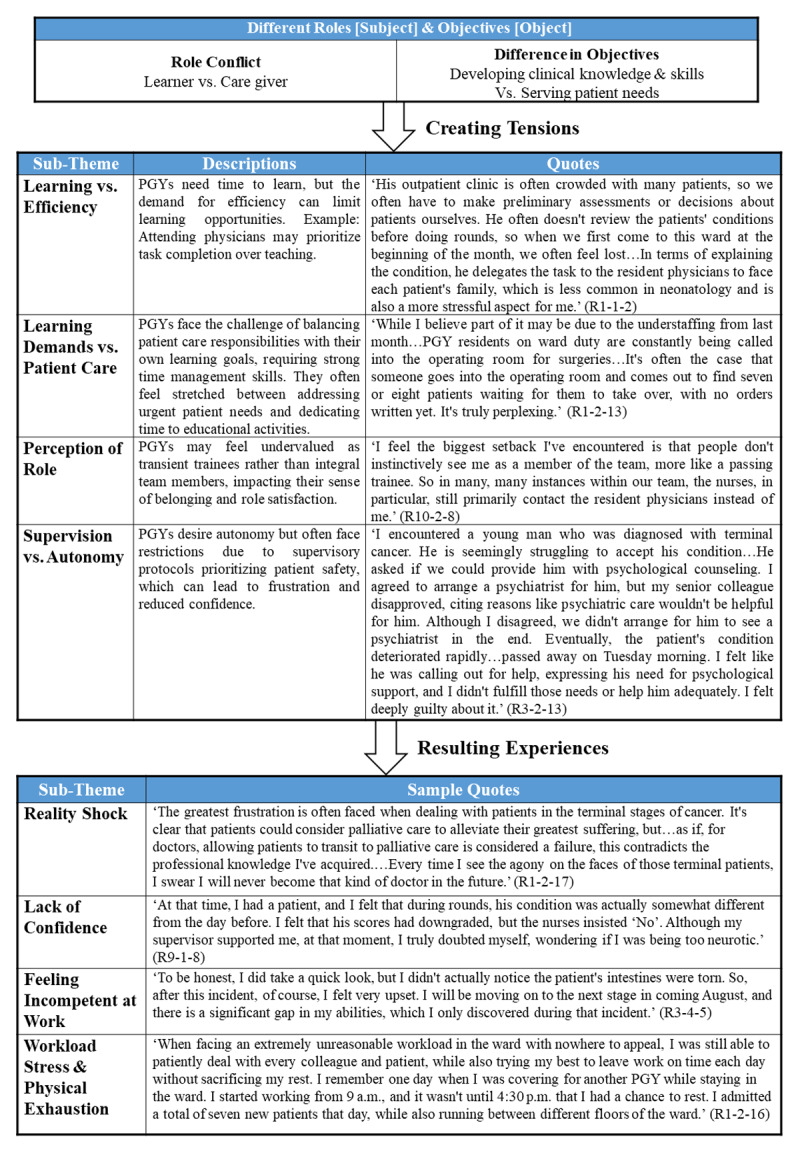
Role conflicts create contradictions/tensions. The quotations are labeled as (participant number; record number; quotation number).

### Resulting experiences

The interactions and conflicts arising from these roles and contradictions give rise to various challenges and frustrations for PGYs. One common obstacle is the experience of reality shock, where PGYs find themselves confronted with a stark contrast between their expectations and the actual realities of clinical practice. This disparity can be disorienting and unsettling, requiring a significant adjustment period. Additionally, many PGYs grapple with a lack of confidence, harboring doubts about their abilities and what they can contribute to patient care. This uncertainty can undermine their self-assurance and hinder their professional growth. Moreover, feelings of incompetence at work can arise, leading to frustration and a sense of inadequacy in fulfilling their responsibilities. These challenges are compounded by the immense workload and physical exhaustion that PGYs often face. Balancing demanding work schedules, long shifts, and heavy patient loads can take a toll on their physical and mental well-being, leaving them feeling drained and fatigued. Collectively, these obstacles highlight the importance of providing support and resources to help PGY interns navigate these challenges effectively and thrive in their roles as healthcare professionals. The main resulting experiences and example quotes are presented in Table 1.

## Discussion

Our study adds valuable insights to the existing literature on the learner-to-doctor transition by highlighting the longitudinal progression of PGY interns as they navigate this role tension over time. Findings reveal that the conflict between learner and doctor roles results in various experiences. Recommendations include improving communication and support structures in training to better align educational objectives with compassionate care. These insights build on existing studies, such as Carlsson et al. (2023) [33], which focused on the internal (psychological) meaning that novice doctors associate with the internship period; our findings provide empirical evidence of the experiences and learning interactions that novice doctors have within the external community of practice. This study’s strength lies in its two-year longitudinal design, which allowed for an in-depth exploration of the evolving PGY internship experience.

Using Activity Theory as an analytical framework helped us examine the intricate dynamics shaping the PGY experience, particularly the tension between learner and doctor roles. Findings highlight the impact of these role tensions on professional identity formation and emphasize the need to enhance communication and support systems within training programs. While previous researchers have examined contradictions in medical education, they have not explicitly delved into the PGY experience [34,35]. This study uncovered prevalent role conflicts and contradictions as PGYs grappled with balancing their educational objectives with the demands of patient care. These conflicts often led to challenges and frustrations, including reality shock, feelings of incompetence, and the stress of managing heavy workloads, aligning with findings from earlier studies [9,36]. By recognizing and addressing the multifaceted challenges faced by PGY interns, strategies can be devised to enact changes that bolster their professional development and well-being, ultimately ensuring the delivery of high-quality healthcare and nurturing the next generation of healthcare professionals.

Stakeholders within the activity systems are in the best position to identify areas for improvement, design appropriate strategies, and implement changes to bring about transformation [30]. It is imperative to devise strategies that not only mitigate these conflicts but also foster the learners’ professional growth and well-being. One promising approach is to leverage the fundamental principles of the Symbiotic Clinical Model, while also considering the elements outlined in Activity Theory: Tools, Rules, and Division of Labor. The concept of Symbiosis in clinical education had previously been raised [37]. However it was the work of Worley and Prideaux that developed the symbiotic clinical education model [38]. The model highlights the importance of achieving ‘symbiosis’ or mutual benefit, where clinical education enhances and takes place within the framework of clinical practice, healthcare delivery, and personal as well as professional growth [38]. Central to a symbiotic clinical model is ensuring reciprocal relationships that yield mutual benefits and strive for a mutually beneficial arrangement among all stakeholders [38,39]. Analysis of participants’ reflections revealed recurring themes of interdependence and shared responsibility between PGYs and supervising doctors, as well as among the broader healthcare team. These relationships indicate that achieving quality patient care and positive health outcomes requires a “symbiotic” relationship, where the growth and success of each role support and strengthen the others. For instance, as PGYs gain critical skills and competencies from supervisors, their evolving capabilities, in turn, enhance the team’s capacity to deliver holistic care. With a clearer understanding about the tensions and contradictions in activity systems, the knowledge shed lights on how to develop strategies for cultivating a collaborative learning environments to support PGY trainee development.

Bringing changes to the PGY journey ensures its fulfillment and entirety. Expansive learning, a component of activity theory aimed at driving change, has been developed and applied in many studies since its introduction by Engeström in 1987 [40]. It involves a collective process where PGYs question, analyze, and test new approaches, creating cycles of reflection and adaptation that ultimately reshape professional practices [18]. Expansive Learning is triggered by reflecting on contradictions within the system, leading to developmental changes. As contradictions are recognized and resolved, both roles and activity systems undergo qualitative transformations [19]. A large-scale cycle of expansive learning develops over years and implies medium cycles, which are composed of small cycles that could occur in a couple of hours. Expansive learning results in a reexamination of the activity’s purpose, nature, and beneficiaries, empowering practitioners through this rediscovery [41].

A review of the existing literature provides recommendations for change, organized according to the components of the activity system and summarized in Table 2. For example, fostering compassionate physicians requires structured opportunities for role modeling alongside the application of expansive learning principles. Through the tool of role modeling, experienced clinicians demonstrate empathy, patient-centered communication, and active listening, offering PGYs real-life examples of compassionate care. Observing these behaviors enables PGYs to internalize and embody compassionate values, promoting their development beyond technical clinical skills. This approach is further strengthened by expansive learning, which encourages PGYs to reflect on and resolve tensions within their roles, sparking transformative changes in their professional identity [19].

**Table 2:** Recommendations for change.


KEY COMPONENTS	RECOMMENDATIONS (REFERENCES)

**1. Tools**	1.1 Utilize Formal Mentorship Programs (Graves, J 2022; Abrams, M.P. 2022 [42,43]) 1.2 Establish Peer Support Networks (Klein, Harrison J 2022; Challa, K.T. 2021 [41,44]) 1.3 Introduce Wellness Initiatives (Lasitha Samarakoon, T 2013 [45]) 1.4 Incorporate Flexible Learning Methods (Tan, X.H; 2021; K.K. Shyamala 2022 [46,47]) 1.5 Provide Comprehensive Communication Skills Training (Bing-You, R 2017; Stamm, M 2011 [48,49]

**2. Rules**	2.1 Establish Feedback and Reflection Process (Deb, Lena 2022 [50]) 2.2 Offer Dedicated Support Services (Lasitha Samarakoon, T 2013 [45])

**3. Division of Labor**	3.1 Promote Interprofessional Collaboration (Engestrom, Y 1995; Carlsson, Y 2023 [32,33]) 3.2 Encourage Learner Engagement (Bendowska, A 2023 [51]) 3.3 Create Supportive Learning Environment (Burgess, A 2018 [52])


Developing new forms of activity systems with the recommendations can support the professional development and well-being of PGYs. In clinical settings, fostering a symbiotic environment externally, while promoting an expansive learning process internally, could lead to new work activity patterns that benefit both PGYs and the broader community. Future research might focus on fostering professional growth and wellness by implementing the proposed strategies in the learning and working environments of PGYs.

## Limitations

While this study offers valuable insights into the PGY internship experience, several limitations should be acknowledged. Firstly, the small sample size of 10 participants may limit the generalizability of the findings to a broader population of PGYs. However, by utilizing repeated collection of recordings, the textual records reached a total of 35 pieces, which is an acceptable amount of data for qualitative analysis. Another limitation of this study is the potential for selection bias, as participants volunteered to participate. This voluntary involvement might lead to a sample that does not fully represent all PGY interns, as those who opted to participate may possess distinct perspectives or experiences compared to those who did not.

## Conclusions

In conclusion, this longitudinal qualitative analysis utilizing Activity Theory highlighted the challenges inherent in balancing educational objectives with clinical responsibilities. The analysis of activity systems revealed the complex interactions and dynamics shaping the PGY experience, with conflicts arising from the tension between the roles of learners and healthcare providers. These role conflicts gave rise to challenges such as reality shock, feelings of incompetence, and the stress of managing heavy workloads. Moving forward, it is essential to build upon these findings and develop tailored interventions to support the professional development and well-being of PGYs. By fostering a symbiotic environment externally, while promoting an expansive learning process internally, healthcare organizations can better prepare PGYs for the demands of clinical practice while promoting a culture of continuous learning and growth.

## Additional File

The additional file for this article can be found as follows:

10.5334/pme.1499.s1 Appendix 1. Audio Diary Questions.
