# Supplementary material for: From Learner to Provider: Navigating Role Tensions in Postgraduate Medical Training Through Activity Theory
Source: Perspect Med Educ. 2025 Feb 14;14(1):55–65. doi: 10.5334/pme.1499 (PMC11827561; doi:10.5334/pme.1499)
Supplement: Appendix 1. — Audio Diary Questions. [file pme-14-1-1499-s1.pdf]

Appendix 1. Audio Diary Questions

Please answer the following questions in an audio format.

1. Please choose one department where you have rotated in the past two months and share your experience.
   - Briefly describe the different members of the teaching team in your rotation (attending physicians, nurses, specialists, etc.) (*please do not mention names*) and how they interacted with you (*please do not mention names*).
   - What was your role and contribution in this team? Please provide examples.
2. What do you think has been your greatest improvement in the past two months? Please provide one aspect and one example.
3. What have been your biggest frustration in the past two months?

Note: Your responses will be kept strictly confidential, and all data will be securely stored to protect your privacy. Please feel free to record your thoughts, as this information will be used only for research purposes.
